# Zinc-Related Proteasome Variants in Type 1 Diabetes: An in Silico-Guided Case-Control Study

**DOI:** 10.3390/metabo15120772

**Published:** 2025-11-28

**Authors:** Raif Gregorio Nasre-Nasser, Anna Carolina Meireles Vieira, Felipe Mateus Pellenz, Luciane Moretto, Eliandra Girardi, Taís Silveira Assmann, Chih-Hao Lu, Luís Henrique Canani, Cristine Dieter, Daisy Crispim

**Affiliations:** 1 Graduate Program in Medical Sciences: Endocrinology, Faculty of Medicine, Department of Internal Medicine, Universidade Federal do Rio Grande do Sul, Porto Alegre 90035-000, Rio Grande do Sul, Brazil; raifgnn@gmail.com (R.G.N.-N.);; 2 Endocrine Division, Hospital de Clínicas de Porto Alegre, Porto Alegre 90035-003, Rio Grande do Sul, Brazil; 3 Escola da Saúde, Universidade do Vale do Rio dos Sinos—UNISINOS, São Leopoldo 93022-154, Rio Grande do Sul, Brazil; 4 Institute of Bioinformatics and Systems Biology, National Yang Ming Chiao Tung University, Hsinchu 300093, Taiwan; 5 Programa de Pós-Graduação em Saúde e Desenvolvimento Humano, Universidade La Salle, Canoas 92010-000, Rio Grande do Sul, Brazil

**Keywords:** T1DM, proteasome, single nucleotide polymorphism, zinc, *PSMC6*, *PSMB9*, *PSMA6*, *PSMD3*, kidney disease, retinopathy

## Abstract

Introduction: The proteasome is a multicatalytic complex responsible for protein degradation and regulation of immune responses, and has been implicated in type 1 diabetes mellitus (T1DM) pathogenesis. Zinc (Zn^2+^) is essential for insulin granule biogenesis and modulates proteasomal activity. This study investigated associations between single-nucleotide polymorphisms (SNPs) in proteasomal subunits predicted to bind Zn^2+^ and T1DM susceptibility or related traits. Methods: This case–control study included 654 patients with T1DM and 573 subjects without DM from Southern Brazil. SNPs were selected through in silico analysis using MIB docking platform to identify Zn^2+^-interacting residues in proteasomal subunits. Five SNPs in proteasomal subunit genes—*PSMA6* (rs1048990), *PSMB6* (rs2304975), *PSMB9* (rs17587), *PSMC6* (rs2295825), and *PSMD3* (rs3087852)—were genotyped using TaqMan assays. Results: The *PSMC6* rs2295825C allele was associated with lower T1DM (OR = 0.77, 95% CI 0.61–0.97; *p* = 0.028) and diabetic retinopathy (DR; OR = 0.65; 95% CI 0.42–0.99; *p* = 0.048) risk, and a more favorable lipid profile (higher HDL-C, lower triglycerides) compared to the G/G genotype. The *PSMB9* rs17587A/A genotype was linked to higher total cholesterol and HbA1c levels. The *PSMA6* rs1048990G allele was linked to increased prevalence of diabetic kidney disease (DKD; OR = 1.75, 95% CI 1.02–2.99; *p* = 0.042), and the *PSMD3* rs3087852A allele was associated with lower urinary albumin excretion. No significant associations were observed for the *PSMB6* rs2304975 SNP. Conclusions: The *PSMC6* rs2295825 SNP may confer protection against T1DM. The *PSMC6* rs2295825, *PSMB9* rs17587, *PSMA6* rs1048990, and *PSMD3* rs3087852 SNPs appear to influence lipid metabolism and diabetic microvascular complications.

## 1. Introduction

The proteasome is a multiprotein complex essential for degrading damaged or unnecessary proteins, playing a central role in maintaining cellular homeostasis and regulating key processes such as the cell cycle, immune response, and apoptosis [1,2]. It contains a catalytic core particle (20S) and one or two regulatory particles (19S or 11S) that modulate its function [3]. The 20S core particle is composed of four stacked heptameric rings, comprising 14 distinct subunits with caspase-like, trypsin-like, and chymotrypsin-like hydrolytic activities [2,4]. The 19S regulatory particle contains 19 different subunits with ATPase and non-ATPase activities, which are responsible for recognizing, unfolding, and translocating substrates into the proteolytic chamber by opening the entry channel [2,4]. Together, the 20S core and 19S particle assemble into the 26S proteasome, which accounts for approximately 2% of total cellular protein content [2].

Additionally, specific isoforms of the proteasome—such as the immunoproteasome (expressed in immune cells) and the thymoproteasome (restricted to the thymus)—play specialized roles in antigen presentation and T-cell selection, respectively [1,2]. Type 1 diabetes mellitus (T1DM) is an autoimmune disease characterized by insulin deficiency resulting from T-cell-mediated destruction of pancreatic beta-cells [5]. In this context, it has been proposed that the misfolding of proinsulin and other secretory proteins in pancreatic cells, particularly in association with endoplasmic reticulum-associated degradation pathways, may lead to their processing by the proteasome [6]. This proteasomal degradation can increase presentation of autoantigenic peptides to immune cells, thereby amplifying the autoimmune response [6].

Single-nucleotide polymorphisms (SNPs) in proteasome subunit genes have been associated with T1DM and other autoimmune diseases [7]. Within the regulatory subunits, the rs2295826 and rs2295827 SNPs in the *PSMC6* (RPT4) gene were associated with T1DM in a Latvian population [8]. Similarly, the rs4065321 and rs709592 SNPs in the *PSMD3* (RPN3) were associated with glucose-related traits and insulin resistance, with evidence suggesting that these associations may be modulated by dietary factors [9]. In the catalytic subunit, the rs17587 SNP in the *PSMB9* (β1i) gene—resulting in an arginine to histidine substitution at position 60 (Arg60His)—has been identified as a risk factor for T1DM in both Asian and Caucasian populations carrying the HLA DR4-DQB1*0302 haplotype [10,11]. In addition, the rs2277460 SNP in the *PSMA6* (α1) gene has been linked to T1DM as well as other metabolic disorders, including type 2 diabetes mellitus (T2DM) and coronary artery disease [8,12].

Zinc (Zn^2+^) is an essential micronutrient that plays a critical role in insulin synthesis, storage, and secretion, as well as in modulating proteasome activity. At elevated concentrations, Zn^2+^ can inhibit both ubiquitination and proteasome-mediated protein degradation, thereby affecting cellular proteostasis [13]. Studies have demonstrated that Zn^2+^ inhibits the proteolytic activity of the 20S proteasome, possibly by displacing magnesium ions (Mg^2+^) from their binding sites within the catalytic chamber [14]. Furthermore, Zn^2+^ regulates the expression and turnover of zinc transporters, which are internalized and degraded via the proteasome in response to fluctuations in Zn^2+^ and other metal ion concentrations [15]. This complex interplay between Zn^2+^ homeostasis and proteasomal function may contribute to the pathogenesis of T1DM.

Given this background, the aim of this study was to investigate the association between SNPs in proteasome subunit genes containing predicted Zn^2+^-binding sites and susceptibility to T1DM, using an approach that integrates in silico predictions with molecular analyses in a case–control study.

## 2. Materials and Methods

### 2.1. Study Population and Sample Size

This multicenter case–control study was conducted in accordance with the STROBE and STREGA guidelines for reporting genetic association studies [16,17]. A total of 654 patients with T1DM were recruited from outpatient clinics at the Hospital de Clínicas de Porto Alegre (HCPA) and the Instituto da Criança com Diabetes—Grupo Hospitalar Conceição (Porto Alegre, Rio Grande do Sul, Brazil) between January 2005 and March 2023 [16,17,18]. T1DM diagnosis was confirmed based on the criteria established by the American Diabetes Association [19]. The control group consisted of 573 individuals without diabetes recruited from the HCPA Blood Bank. Controls were selected based on the absence of a family history of diabetes (in first-degree relatives) and glycated hemoglobin (HbA1c) levels below 5.7%. The study protocol was approved by the HCPA Research Ethics Committee (approval number: 58089422.9.0000.5327), and all participants provided written informed consent prior to enrollment.

The sample size was calculated using the PSS Health platform [20], with a significance level (α) of 0.05 and statistical power (β) of 80%. Calculations were based on the minor allele frequencies of the selected SNPs, obtained from the 1000 Genomes Project database (https://www.ncbi.nlm.nih.gov/snp/ (accessed on 5 February 2022)), and assumed effect sizes [odds ratios (OR) of 0.5 for protective alleles and 1.5 for risk alleles] [8,9,10,11,21].

### 2.2. Clinical and Laboratory Evaluations

All participants underwent a comprehensive evaluation, including a medical interview, physical examination, and blood collection for laboratory analysis and DNA extraction, as previously described [16,17,18]. Collected data included demographic information (age, sex, and self-reported ethnicity), anthropometric measurements [height, weight, and body mass index (BMI = kg/m^2^)], hypertension status, age at T1DM diagnosis, and diabetes duration at the time of sample collection. Laboratory analyses were performed using standardized techniques. Blood glucose levels were measured using the glucose oxidase-peroxidase coupled reaction, and HbA1c was quantified by ion-exchange high-performance liquid chromatography (HPLC), calibrated to the Diabetes Control and Complications Trial (DCCT) standard [22]. Lipid profile—total cholesterol, high-density lipoprotein cholesterol (HDL-C), and triglycerides—was assessed using enzymatic colorimetric methods. Low-density lipoprotein cholesterol (LDL-C) was calculated using the Martin et al. formula [23]. Serum creatinine levels were determined by the Jaffé colorimetric method, and urinary albumin excretion (UAE) was measured by immunoturbidimetry.

Diabetic kidney disease (DKD) was diagnosed based on the Kidney Disease Improving Global Outcomes (KDIGO) guidelines [24], using UAE levels and the estimated glomerular filtration rate (eGFR), calculated using the Chronic Kidney Disease Epidemiology Collaboration (CKD-EPI) equation [25]. Following these criteria, DKD was classified as absent in individuals with at least 10 years of T1DM duration, eGFR ≥ 90 mL/min/1.73 m^2^, and UAE < 30 mg/g, and as present in those with eGFR < 90 mL/min/1.73 m^2^ and/or UAE ≥ 30 mg/g. Diabetic retinopathy (DR) was evaluated by an experienced ophthalmologist through fundoscopy with pupil dilation, following the criteria established by the Global Diabetic Retinopathy Project Group [26].

### 2.3. In Silico Analysis and Selection of SNPs of Interest

SNPs were selected based on their predicted interaction with Zn^2+^ in proteasome subunits analyzed using the Metal Ion-Binding (MIB) docking platform (http://bioinfo.cmu.edu.tw/MIB/ (accessed on 17 July 2022)) [27]. MIB employs a structure-based approach to identify metal ion binding sites in proteins, applying geometric and energetic criteria to predict interactions between Zn^2+^ ions and amino acid residues of the proteasome. Initially, ten proteasome subunits were pre-selected based on previously reported associations with T1DM or other autoimmune/metabolic diseases [7,8,9,10,11,12,21,28]. The analysis included six subunits from the 20S core particle, based on crystallographic 3D structures available in the Protein Data Bank [PDB (https://www.rcsb.org (accessed on 13 June 2022)): 5LE5 and 7AWE]: α1 (*PSMA6*), α7 (*PSMA3*), β1 (*PSMB6*), β1i (*PSMB9*), β5 (*PSMB5*), and β5i (*PSMB8*). In addition, four subunits from the 19S regulatory particle were considered, using the crystallographic structure of the 26S complex (PDB: 6MSB): RPT4 (*PSMC6*), RPN3 (*PSMD3*), RPN10 (*PSMD4*), and RPN11 (*PSMD14*). Protein domains containing Zn^2+^-binding sites were ranked according to the number and scores of potential interaction sites. Genomic regions encoding these domains were then analyzed to identify SNPs with a minor allele frequency greater than 10%. Priority was given to SNPs located in exonic regions of domains with Zn^2+^-binding sites—namely rs2304975 (C/T) in *PSMB6*, rs17587 (G/A) in *PSMB9*, and rs3087852 (G/A) in *PSMD3*—or to informative variants in linkage disequilibrium (LD) with target regions, including rs1048990 (C/G) in *PSMA6* and rs2295825 (G/C) in *PSMC6*. LD analysis was performed using the LDlink platform (https://ldlink.nci.nih.gov/?tab=ldpop (accessed on 21 August 2022)) and we selected SNPs with D’ > 0.8 with informative SNPs in reference populations from the 1000 Genomes Project. SNPs with MAF < 10% or not satisfying the LD threshold were excluded from further analyses.

### 2.4. Molecular Analyses

Total DNA was extracted from peripheral blood leukocytes using the FlexiGene^®^ DNA Kit (Qiagen, Germantown, MD, USA), according to the manufacturer’s instructions. Genotyping was conducted using allelic discrimination real-time PCR with TaqMan^®^ SNP Genotyping Assays (Thermo Fisher Scientific, Foster City, CA, USA). Reactions were performed in 384-well plates with a final volume of 5 µL, containing 2 ng of DNA, TaqMan^®^ ProAmp Master Mix, and the specific TaqMan^®^ SNP Genotyping Assay (see Table 1 for SNPs and assay details). The thermal cycling protocol consisted of an initial denaturation at 95 °C for 10 min, followed by 50 cycles at 95 °C for 15 s and 62 °C for 90 s. Negative controls were included in all runs to ensure assay specificity. The overall genotyping success rate exceeded 95%, and reproducibility was verified by re-genotyping 10% of the samples, yielding 100% concordance.

### 2.5. Statistical Analyses

Genotype frequencies for each SNP were tested for compliance with Hardy–Weinberg equilibrium using the χ^2^ goodness-of-fit test. Allele and genotype frequencies were compared between groups using χ^2^ tests, and genotype distributions were analyzed under additive, recessive, and dominant inheritance models [29]. The effect size of each SNP on T1DM was estimated using odds ratios (OR) with 95% confidence intervals (CI). Continuous variables were assessed for normality using the Kolmogorov–Smirnov test. Parametric data were analyzed using independent one-way ANOVA or *t*-tests, as appropriate. Non-normally distributed variables were analyzed using non-parametric methods. Logistic regression analyses were performed to evaluate the independent associations between SNPs and T1DM, adjusting for potential covariates. Linear regression was used to assess associations between SNPs and normally distributed continuous variables, while generalized linear models (GLM) with Gamma distribution and log link function were applied for variables with non-normal distribution, both adjusting for confounders. For these analyses, model choice was based on residual distribution and goodness-of-fit. Results from linear regression and GLM are reported as adjusted mean differences (B) or adjusted geometric mean [Exp(B)] with corresponding 95% CI. Data are presented as mean ± standard deviation (SD), median (25th–75th percentiles), or percentages, as appropriate. Statistical significance was defined as *p* < 0.05. All statistical analyses were conducted using SPSS software version 18.0 (SPSS Inc., Chicago, IL, USA).

## 3. Results

### 3.1. Sample Description

The study included 654 individuals with T1DM and 573 controls without diabetes. Demographic and clinical characteristics are summarized in Table 2. Compared to T1DM group, the control group was older (mean age: 37.2 ± 10.7 vs. 34.6 ± 12.3 years, *p* < 0.0001) and had a higher mean BMI (27.1 ± 4.5 vs. 24.8 ± 4.0 kg/m^2^, *p* < 0.0001). A greater proportion of females was observed in the T1DM group compared to controls (51.5% vs. 44.1%, *p* = 0.011), while self-identified Black individuals were more prevalent among controls (14.4% vs. 9.2%, *p* = 0.005). Among T1DM patients, the mean age at diagnosis was 14.8 ± 9.1 years, and the mean duration of diabetes was 18.8 ± 9.2 years. DKD was present in 37.1% of patients, while DR was observed in 50.3% of them. Additionally, systemic arterial hypertension was observed in 37.4% of individuals with T1DM.

### 3.2. Predicted Zn^2+^-Binding Sites in Proteasome Subunits

**Figure 1:**
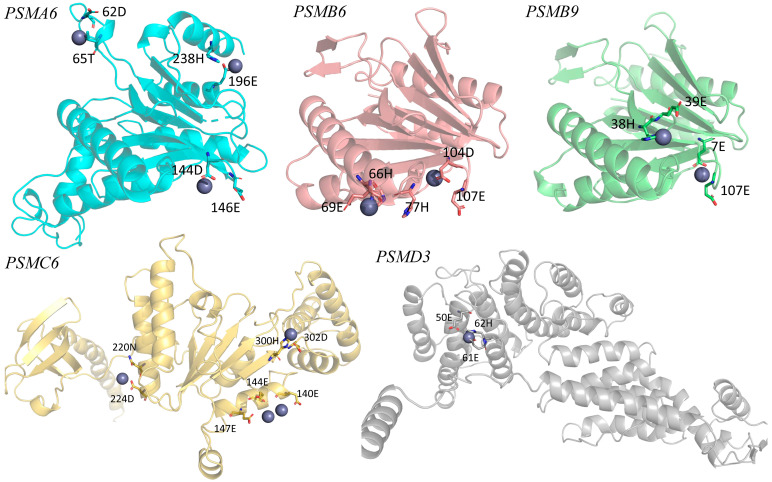
Predicted Zn^2+^-binding sites in proteasome subunits. Subunits *PSMA6*, *PSMB6*, *PSMB9*, *PSMC6*, and *PSMD3* are shown with the residues corresponding to the main Zn^2+^-binding sites predicted using the MIB platform. Residues are indicated by the residue number in the protein sequence followed by the one-letter amino acid code (e.g., 62D corresponds to aspartic acid at position 62 in *PSMA6*), according to the IUPAC/IUBMB standard nomenclature for amino acids (https://www.qmul.ac.uk/sbcs/iubmb/ (accessed on 20 July 2022)). These sites correspond to the interaction hotspots identified in the docking analysis.

The docking analysis predicted multiple Zn^2+^-binding residues across the proteasome subunits, with scores ranging from approximately 1.9 to 8.4 (Table S1). In the 20S core particle, relevant interaction hotspots were identified in *PSMA6* (62D, 65T, 144D, 146E, 196E, and 238H), *PSMB6* (66H, 69E, 77H, 104D, and 107E), and *PSMB9* (7E, 38H, 39E, and 107E). Within the 19S regulatory particle, *PSMC6* presented several predicted Zn^2+^-binding sites (140E, 144E, 147E, 220N, 224D, 300H and 302D), and *PSMD3* displayed the highest overall docking scores, particularly at 50E, 61E and 62H (Figure 1).

Although additional residues with potential Zn^2+^ interaction were detected in other subunits, most of the corresponding genomic variants exhibited minor alleles frequencies below 10%, limiting their suitability for this association study. Therefore, priority was given to informative SNPs located within or in linkage disequilibrium with the identified domains, resulting in the selection of rs1048990 (*PSMA6*), rs2304975 (*PSMB6*), rs17587 (*PSMB9*), rs2295825 (*PSMC6*), and rs3087852 (*PSMD3*) for further genotyping in our samples.

### 3.3. Genotype and Allele Distributions in Patients with T1DM and Individuals Without Diabetes

All analyzed SNPs were in Hardy–Weinberg equilibrium in the control group (*p* > 0.050). Genotype and allele frequencies of the proteasome-related SNPs analyzed in patients with T1DM and controls are summarized in Table 3. Genotype distributions of the *PSMC6* rs2295825 SNP showed a significant difference between T1DM and control groups (*p* = 0.046). The presence of the C allele (G/C + C/C) of rs2295825 SNP was more prevalent among controls than among patients with T1DM (61.0% vs. 55.1%, *p* = 0.044). After adjusting for sex and ethnicity, the presence of the C allele remained associated with protection against T1DM (OR = 0.772, 95% CI 0.613–0.973; *p* = 0.028 for the dominant model).

For the remaining SNPs, no significant differences in genotype or allele frequencies were observed between patients with T1DM and controls, even after adjustment for sex and ethnicity (all adjusted *p* values > 0.050; Table 3). Specifically, the frequency of the G allele of *PSMA6* rs1048990 SNP was 18.4% in patients with T1DM and 17.1% in controls (*p* = 0.423), while the T allele of *PSMB6* rs2304975 SNP was detected in 7.8% of cases and 8.0% of controls (*p* = 0.850). The A allele of *PSMB9* rs17587 SNP appeared in 29.3% of cases vs. 26.9% of controls (*p* = 0.222), and the A allele of *PSMD3* rs3087852 SNP was found in 49.0% of cases compared to 50.3% of controls (*p* = 0.533).

### 3.4. Allele Distribution in Clinical and Laboratory Characteristics of T1DM

In an exploratory analysis, the associations between the analyzed SNPs and clinical or laboratory characteristics of T1DM were evaluated (Table S2). For the *PSMC6* rs2295825 SNP, carriers of the C allele showed significantly higher HDL-C levels compared to non-carriers (G/C + C/C: 58.3 ± 16.8 mg/dL vs. G/G: 54.4 ± 16.9 mg/dL; *p* = 0.012, dominant model). This association remained statistically significant after adjustment for sex, age, and BMI (B = 3.583; 95% CI: 0.384–6.782; *p* = 0.028 from linear regression). Triglyceride levels were significantly lower among carriers of the C allele [G/C + C/C: 75.5 mg/dL (58.8–110.3) vs. G/G: 89.5 mg/dL (58.0–136.5); *p* = 0.019]. This association persisted after adjustment for sex, age, and BMI, indicating that C allele carriers had 16.6% lower triglyceride levels compared to non-carriers [Exp(B) = 0.834; 95% CI: 0.740–0.939; *p* = 0.003 from Gamma GLM]. Furthermore, C allele (G/C + C/C genotypes) carriers had a lower prevalence of DR compared to those with the G/G genotype (45.3% vs. 56.5%, *p* = 0.009). This association remained statistically significant after adjustment for age, T1DM duration, hypertension, and serum creatinine (OR = 0.650; 95% CI: 0.423–0.997; *p* = 0.048).

The *PSMB9* rs17587 SNP was associated with total cholesterol levels, with individuals carrying the A/A genotype showing significantly higher levels compared to those with the G/G and G/A genotypes (203.4 ± 62.6 mg/dL vs. 180.9 ± 47.6 mg/dL; *p* = 0.035, recessive model). This association remained significant after adjusting for sex, age, and BMI (B = 23.896; 95% CI: 5.964–41.828; *p* = 0.009 from linear regression). Although the *PSMB9* rs17587 A/A genotype was also associated with higher HDL cholesterol levels in the crude analysis (62.5 ± 17.6 mg/dL vs. 56.1 ± 17.0 mg/dL; *p* = 0.030), this association was not maintained after adjustment for sex, age, and BMI (B = 3.112, 95% CI: −2.783–9.006; *p* = 0.300 from linear regression). Moreover, the A/A genotype was associated with higher HbA1c levels compared to the presence of the G allele (9.5 ± 2.0% vs. 8.8 ± 2.0%, *p* = 0.045, recessive model).

For the *PSMA6* rs1048990 SNP, carriers of the G allele (C/G + G/G genotypes) exhibited a higher prevalence of DKD compared to those with the C/C genotype (43.8% vs. 33.4%; *p* = 0.049, dominant model). This association remained significant after adjustment for HbA1c, T1DM duration, hypertension, and presence of DR (OR = 1.747, 95% CI: 1.020–2.992; *p* = 0.042). Regarding the *PSMD3* rs3087852 SNP, A allele carriers (G/A + A/A genotypes) showed lower UAE than those with the G/G genotype [9.5 mg/g (4.8–40.5) vs. 19.0 mg/g (5.0–196.4), *p* = 0.016, dominant model], and this association persisted after adjustment for HbA1c, T1DM duration, hypertension, and presence of DR [Exp(B) = 0.640; 95% CI: 0.441–0.930; *p* = 0.019 from Gamma GLM]. No significant associations were found between the *PSMB6* rs2304975 SNP and any clinical or laboratory characteristics in the T1DM group.

## 4. Discussion

This case–control study investigated SNPs in proteasomal subunits containing predicted Zn^2+^-binding sites in a Southern Brazilian population with T1DM, using a combination of in silico and molecular approaches. Docking analysis identified candidate residues for Zn^2+^ interaction across multiple subunits, with scores ranging from 1.9 to 8.4, which guided the selection of SNPs for genetic association testing. Among the five SNPs analyzed, only *PSMC6* rs2295825 showed a significant association with T1DM, with the C allele conferring a protective effect. The other SNPs—*PSMA6* rs1048990, *PSMB6* rs2304975, *PSMB9* rs17587, and *PSMD3* rs3087852—were not significantly associated with T1DM risk. However, exploratory analyses revealed allele-dependent differences in metabolic traits and microvascular complications, suggesting potential pleiotropic effects of these SNPs.

*PSMC6*, also known as RPT4, encodes an ATPase subunit of the 19S regulatory particle, which is essential for substrate unfolding and translocation into the 20S core for proteasomal degradation [4]. The rs2295825 SNP is located within the first intron of the *PSMC6* gene [30], and emerged as a relevant variant in our study, with the C allele being associated with a protective effect against T1DM (OR = 0.772, *p* = 0.028). Although the rs2295825 SNP has not been previously investigated in the context of T1DM, we prioritized it based on its minor allele frequency and strong LD (D’ = 1.0) with two neighboring SNPs—rs2295826 and rs2295827—that have been implicated in autoimmune diseases, including T1DM [8,31,32]. Intriguingly, the C allele of rs2295825 SNP was reported to be in LD with known T1DM risk alleles of the rs2295826–rs2295827 haplotype (OR = 1.765) [8], suggesting either a haplotype inversion or distinct gene–environment interactions across populations. A similar population-specific LD shift was observed in Taiwanese versus Latvian cohorts, underscoring the importance of ethnic context in interpreting proteasome-related SNPs [30].

Functional in silico analyses suggest that the *PSMC6* rs2295826 and rs2295827 SNPs are located in critical splicing regions and branch points, potentially affecting alternative splicing events. These SNPs also lie within sequences recognized by transcription factors, including the cyclic AMP response element-binding protein (CREB) [30]. Mechanistically, CREB is a well-established transcriptional activator involved in key metabolic pathways, including hepatic gluconeogenesis, lipogenesis, and pancreatic beta-cell function [33]. It has also been implicated in DR via cAMP/PKA and CaMK2A signaling, contributing to retinal neurodegeneration [34,35]. *PSMC6* itself acts as a hub proteasomal subunit involved in oxidative stress responses in retinal cells [36]. These functional insights may help explain our findings, in which the *PSMC6* rs2295825 C allele was associated not only with protection against T1DM but also with a favorable metabolic profile, specifically a 3.58 mg/dL increase in HDL-C and a 16.6% reduction in triglyceride levels, as well as protection against DR (OR = 0.65; *p* = 0.048).

*PSMB9* encodes the catalytic subunit β1i, also known as LMP2, which is constitutively expressed in immune cells and can be upregulated by cytokines and interferons [4]. It is a component of both the immunoproteasome and thymoproteasome complexes, where it exhibits caspase-like activity crucial for antigen processing and presentation via HLA class I molecules [1]. Located within the *HLA class II* region, the *PSMB9* gene harbors a functional missense SNP, rs17587, which has been previously associated with an increased risk of autoimmune diseases, including T1DM, particularly in Caucasian and Asian populations [10,11]. In our study, although the rs17587 SNP was not significantly associated with T1DM, patients with T1DM with the A/A genotype exhibited higher HbA1c levels compared to G allele carriers (9.5 ± 2.0% vs. 8.8 ± 2.0%, *p* = 0.045), suggesting a potential impact of this SNP on glycemic control. The lack of a direct association with T1DM may reflect ethnic-specific effects or context-dependent modulation of *PSMB9* function.

Although the *PSMB9* rs17587 SNP was not associated with T1DM in our population, individuals with the A/A genotype exhibited significantly higher total cholesterol levels (+23.9 mg/dL; *p* = 0.009), suggesting a potential metabolic effect that may be independent of classical immune regulation. While the underlying mechanisms of this association remain unclear, emerging evidence implicates *PSMB9* in the regulation of lipid metabolism and vascular inflammation [37]. In the *Apoe*^−/−^ mice, *Psmb9* deficiency resulted in smaller atherosclerotic lesions, increased plaque stability, and reduced vascular inflammation, which was mediated through a pathway involving the long noncoding RNA *Psmb8-As1*, the nuclear cofactor NONO, and the transcriptional repressor ZEB1 [37]. Notably, *Psmb9* knockout reversed the pro-atherogenic effects induced by endothelial overexpression of *PSMB8-AS1* [37]. These findings suggest that genetic variation in *PSMB9* may contribute to atherogenic lipid profiles, particularly in individuals with chronic low-grade inflammation.

*PSMA6* encodes the α1 subunit of the proteasome core particle, contributing to the formation of the α-ring that regulates substrate entry into the catalytic chamber [4]. The *PSMA6* rs1048990 SNP is located in the 5’-untranslated region (5’-UTR) of the gene, just eight nucleotides upstream of the start codon, and has been shown to enhance *PSMA6* expression [30]. The rs1048990 SNP has been previously associated with autoimmune diseases such as T1DM, multiple sclerosis, and juvenile idiopathic arthritis [8,31,32], as well as with cardiovascular disease [38,39]. However, no significant association between this SNP and T1DM was observed in our study. This discrepancy may reflect population differences in underlying genetic structure.

Notably, carriers of the *PSMA6* rs1048990 G allele showed an increased risk of DKD, even after adjustment for HbA1c, T1DM duration, hypertension, and presence of DR (OR = 1.747, *p* = 0.042). Interestingly, a previous study in patients with end-stage renal disease (ESRD) suggested a protective effect of this allele; however, only 23% of that cohort had diabetes, limiting the relevance of those findings to DKD [38]. Several functional mechanisms may underlie the association observed in our study, including modulation of NF-κB signaling through IκB phosphorylation [39], altered splicing regulation [30,32], transcriptional control by p53 and DMRT family transcription factors [30,32], and increased promoter methylation at the *PSMA6 locus* [40]. Together, these findings support a potential role for *PSMA6* in the pathogenesis of renal complications in T1DM.

*PSMD3* encodes RPN3, a non-ATPase subunit of the 19S regulatory particle of the proteasome, which is involved in substrate recognition [4]. This is the first study to investigate the association between the *PSMD3* rs3087852 SNP and T1DM, and no direct association was detected. However, other *PSMD3* SNPs have been linked to insulin resistance in different populations, with evidence suggesting that these associations may be modulated by dietary factors [9]. Moreover, the *PSMD3* rs3087852 SNP has previously been associated with circulating levels of resistin [41], an adipokine secreted primarily by mononuclear cells that promotes pro-inflammatory responses and contributes to endothelial dysfunction [42]. Circulating resistin has also been correlated with eGFR and UAE in patients with T2DM or in individuals with hypertension but without DM [43,44,45,46]. In line with these findings, we observed that carriers of the rs3087852 A allele exhibited lower UAE levels compared to those with the G/G genotype.

*PSMB6* encodes the constitutive β1 subunit of the 20S proteasome core, which possesses caspase-like proteolytic activity and plays a key role in protein degradation and immune regulation [4]. This is the first study to evaluate the association between the *PSMB6* rs2304975 SNP and T1DM; however, no significant association was identified. Nevertheless, experimental and transcriptomic evidence support a potential role of PSMB6 in diabetes pathophysiology. In the *NOD* mouse model, high-resolution mapping of the *Idd4* susceptibility locus highlighted *Psmb6* as one of the strongest candidate genes for T1DM [47]. Moreover, transcriptomic profiling of human pancreatic alfa-cells has demonstrated enrichment of proteasome-related genes, including *PSMB6*, suggesting that enhanced proteasomal activity may mitigate endoplasmic reticulum (ER) stress and unfolded protein responses during autoimmunity [48]. Taken together, these findings support a plausible role for *PSMB6* in proteasome-mediated immune and metabolic processes, despite the lack of association between the rs2304975 SNP and T1DM in our study.

This study has several limitations that should be acknowledged. First, although both patients with T1DM and controls were recruited from the same geographic region and healthcare facilities to reduce the risk of population stratification, residual confounding factors cannot be entirely ruled out. Second, while the study was adequately powered to detect moderate effect sizes (OR < 0.5 or >1.5), smaller associations may have gone undetected, introducing the possibility of type II errors. Third, the lack of serum Zn^2+^ measurements limited our ability to explore gene–environment interactions, particularly those involving zinc homeostasis. In addition, most variants predicted to affect Zn^2+^-binding sites had minor allele frequencies < 10% in reference populations and could not be robustly tested in our sample. Therefore, we analyzed tag SNPs in LD with the predicted domains, recognizing their limited ability to reflect causal effects. In addition, we did not assess pancreatic islet autoantibodies (ZnT8A, GADA, IA-2A, and IAA), which could have offered further insight into how the analyzed SNPs relate to autoimmune mechanisms in T1DM. Functional studies and replication in larger and ethnically diverse cohorts are needed to validate the biological relevance of these associations, ideally incorporating serum Zn^2+^ measurements to better elucidate potential gene–environment interactions.

## 5. Conclusions

In summary, our findings support a protective role for the C allele of *PSMC6* rs2295825 SNP in T1DM, alongside additional associations observed between proteasomal SNPs, lipid profile, and microvascular complications (DKD and DR). These results contribute to the growing body of knowledge on the genetic architecture of T1DM and underscore the proteasome complex as a potential link between immune regulation and metabolic homeostasis. Given the established interplay between zinc homeostasis, proteasome function, and diabetes pathophysiology, future studies integrating genomic, transcriptomic, and metabolomic may uncover novel therapeutic targets for autoimmune and metabolic diseases.

## Figures and Tables

**Table 1:** Description of the five analyzed SNPs in *PSMA6*, *PSMB6*, *PSMB9*, *PSMC6*, and *PSMD3* genes.

Gene	Protein Name	SNPs	Alleles	MAF 1000 Genomes	Position (GRCh38.p14)	Region	Assay ID
*PSMA6*	α1/PROS27	rs1048990	C/G	G: 0.1825	chr14: 35292469	5’UTR	C_11599359_10
*PSMB6*	β1/LMPY	rs2304975	C/T	T: 0.1969	chr17: 4797724	Exon	C_15976146_30
*PSMB9*	β1i/LMP2	rs17587	G/A	A: 0.2266	chr6: 32857313	Exon	C_8849004_1
*PSMC6*	RPT4	rs2295825	G/C	C: 0.3173	chr14: 52707610	Intron	C_1310483_30
*PSMD3*	RPN3	rs3087852	G/A	G: 0.4689	chr17: 39981111	Exon	C_16006920_20

Sources: https://www.ncbi.nlm.nih.gov/ (accessed on 20 August 2022), https://www.thermofisher.com/br/ (accessed on 30 August 2022). MAF: minor allele frequency. 5’-UTR: 5’-untranslated region.

**Table 2:** Description of demographic and clinical characteristics of controls without diabetes and patients with T1DM.

Characteristic	Control Group	T1DM Group	*p*-Value *
Age (years)	37.2 ± 10.7	34.6 ± 12.3	<0.0001
Female (%)	44.1	51.5	0.011
Black subjects (%)	14.4	9.2	0.005
HbA1c (%)	5.3 ± 0.3	8.8 ± 1.9	<0.0001
Body mass index (kg/m^2^)	27.1 ± 4.5	24.8 ± 4.0	<0.0001
Age at T1DM diagnosis (years)	-	14.8 ± 9.1	-
T1DM duration (years)	-	18.8 ± 9.2	-
Diabetic kidney disease (%)	-	37.1	-
Diabetic retinopathy (%)	-	50.3	-
Systemic arterial hypertension (%)	-	37.4	-

Values of variables are presented as percentage, mean ± standard deviation. * *p*-value was obtained from *t*-test or χ^2^ test according to each variable.

**Table 3:** Genotype and allele frequencies of SNPs in *PSMA6*, *PSMB6*, *PSMB9*, *PSMC6*, and *PSMD3* genes in patients with T1DM and individuals without diabetes (control group).

Gene/SNP	Control Group	T1DM Group	Non-Adjusted	Adjusted OR (95% CI)/*p*-Value ^§^
			*p*-Value *	
*PSMA6*/rs1048990 (C/G)	(*n* = 573)	(*n* = 654)		
Genotype			0.214	
C/C	394 (68.8)	445 (68.0)		1
C/G	162 (28.3)	177 (27.1)		0.966 (0.748–1.247)/0.788
G/G	17 (3.0)	32 (4.9)		1.795 (0.966–3.337)/0.064
Allele frequency			0.423	-
C	950 (82.9)	1067 (81.6)		
G	196 (17.1)	241 (18.4)		
Dominant model			0.835	
C/C	394 (68.8)	445 (68.0)		1
C/G + G/G	179 (31.2)	209 (32.0)		1.040 (0.815–1.328)/0.752
*PSMB6*/rs2304975 (C/T)	(*n* = 572)	(*n* = 645)		
Genotype			0.88	
C/C	486 (85.0)	550 (85.3)		1
C/T	80 (14.0)	90 (14.0)		1.011 (0.728–1.404)/0.949
T/T	6 (1.0)	5 (0.8)		0.754 (0.228–2.495)/0.643
Allele frequency			0.85	-
C	1052 (92.0)	1190 (92.2)		
T	92 (8.0)	100 (7.8)		
Dominant model			0.945	
C/C	486 (85.0)	550 (85.3)		1
C/T + T/T	86 (15.0)	95 (14.7)		0.784 (0.624–0.986)/0.965
*PSMB9*/rs17587 (G/A)	(*n* = 566)	(*n* = 564)		
Genotype			0.447	
G/G	303 (53.5)	283 (50.2)		1
G/A	222 (39.2)	232 (41.1)		1.123 (0.876–1.440)/0.359
A/A	41 (7.2)	49 (8.7)		1.230 (0.784–1.930)/0.367
Allele frequency			0.222	-
G	828 (73.1)	798 (70.7)		
A	304 (26.9)	330 (29.3)		
Dominant model			0.285	
G/G	303 (53.5)	283 (50.2)		1
G/A + A/A	263 (46.5)	281 (49.8)		1.140 (0.900–1.445)/0.276
Recessive model			0.431	
G/G + G/A	525 (92.8)	515 (91.3)		1
A/A	41 (7.2)	49 (8.7)		1.169 (0.755–1.810)/0.483
Additive model			0.33	
G/G	303 (88.1)	283 (85.2)		1
A/A	41 (11.9)	49 (14.8)		1.226 (0.782–1.923)/0.375
*PSMC6*/rs2295825 (G/C)	(*n* = 569)	(*n* = 655)		
Genotype			0.046	
G/G	222 (39.0)	294 (44.9)		1
G/C	270 (47.5)	265 (40.5)		0.731 (0.572–0.935)/0.013
C/C	77 (13.5)	96 (14.7)		0.917 (0.645–1.305)/0.630
Allele frequency			0.24	-
G	714 (62.7)	853 (65.1)		
C	424 (37.3)	457 (34.9)		
Dominant model			0.044	
G/G	222 (39.0)	294 (44.9)		1
G/C + C/C	347 (61.0)	361 (55.1)		0.772 (0.613–0.973)/0.028
Recessive model			0.631	
G/G + G/C	492 (86.5)	559 (85.3)		1
C/C	77 (13.5)	96 (14.7)		1.075 (0.774–1.494)/0.666
Additive model			0.801	
G/G	222 (74.2)	294 (75.4)		1
C/C	77 (25.8)	96 (24.6)		0.917 (0.645–1.304)/0.630
*PSMD3*/rs3087852 (G/A)	(*n* = 569)	(*n* = 653)		
Genotype			0.577	
G/G	139 (24.4)	176 (27.0)		1
G/A	287 (50.4)	314 (48.1)		0.847 (0.642–1.117)/0.240
A/A	143 (25.1)	163 (25.0)		0.904 (0.656–1.246)/0.539
Allele frequency			0.533	-
G	565 (49.7)	666 (51.0)		
A	573 (50.3)	640 (49.0)		
Dominant model			0.347	
G/G	139 (24.4)	176 (27.0)		1
G/A + A/A	430 (75.6)	477 (73.0)		0.866 (0.667–1.124)/0.279
Recessive model			0.998	
G/G + G/A	426 (74.9)	490 (75.0)		1
A/A	143 (25.1)	163 (25.0)		1.009 (0.775–1.313)/0.949
Additive model			0.568	
G/G	139 (49.3)	176 (51.9)		1
A/A	143 (50.7)	163 (48.1)		0.891 (0.646–1230)/0.484

Values are presented as number (%) or proportion. Recessive and additive inheritance models for the *PSMA6* rs1048990 and *PSMB6* rs2304975 SNPs were excluded from analysis due to low observed allele frequencies. * *p*-values were calculated using χ^2^ tests. **^§^** *p*-values and OR (95% CI) were obtained using logistic regression analyses adjusting for sex and ethnicity.

## Data Availability

All data supporting the findings of this study, including tables and figures, are fully available within the article. In addition, two Supplementary Tables providing further detailed results are available on the journal’s website.

## Supplementary Materials

The following supporting information can be downloaded at: https://www.mdpi.com/article/10.3390/metabo15120772/s1, Table S1: Docking-based prediction of Zn^2+^-binding residues in proteasome subunits; Table S2: Clinical and laboratory characteristics of patients with T1DM stratified by SNPs in *PSMA6*, *PSMB6*, *PSMB9*, *PSMC6*, and *PSMD3* genes.
