# Supplementary material for: Zinc-Related Proteasome Variants in Type 1 Diabetes: An in Silico-Guided Case-Control Study
Source: Metabolites. 2025 Nov 28;15(12):772. doi: 10.3390/metabo15120772 (PMC12734831; doi:10.3390/metabo15120772)
Supplement: Supplementary file 1 [file metabolites-15-00772-s001.zip › metabolites-4000422-supplementary.pdf]

*Article*

# Zinc-Related Proteasome Variants in Type 1 Diabetes: An In Silico-Guided Case-Control Study

Raif Gregorio Nasre-Nasser [1,2], Anna Carolina Meireles Vieira [2], Felipe Mateus Pellenz [2,3], Luciane Moretto [1,2], Eliandra Girardi [2], Taís Silveira Assmann [1], Chih-Hao Lu [4], Luís Henrique Canani [1,2], Cristine Dieter [1,2,5] and Daisy Crispim [1,2,*]

1 Graduate Program in Medical Sciences: Endocrinology, Faculty of Medicine, Department of Internal Medicine, Universidade Federal do Rio Grande do Sul, Porto Alegre, 90035-000, Rio Grande do Sul, Brazil. raifgnn@gmail.com (R.G.N.-N.); lucim.moretto@gmail.com (L.M.); assmann.tais.silveira@gmail.com (T.S.A.); lcanani@hcpa.edu.br (L.H.C.);

2 Endocrine Division, Hospital de Clínicas de Porto Alegre. Porto Alegre, 90035-003, Rio Grande do Sul, Brazil. acmvieira01@gmail.com (A.C.M.V.); flppellenz434@gmail.com (F.M.P.); eggirardi@hcpa.edu.br (E.G.);

3 Escola da Saúde, Universidade do Vale do Rio dos Sinos - UNISINOS, São Leopoldo, 93022-154, Rio Grande do Sul, Brazil.

4 Institute of Bioinformatics and Systems Biology, National Yang Ming Chiao Tung University, Hsinchu, 300093, Taiwan. luchihhao@gmail.com (C.-H.L.)

5 Programa de Pós-Graduação em Saúde e Desenvolvimento Humano, Universidade La Salle. Canoas, 92010-000, Rio Grande do Sul, Brazil. cdieter@hcpa.edu.br

* Correspondence: dcmoreira@hcpa.edu.br

**Table S1.** Docking-based prediction of $Zn^{2+}$-binding residues in proteasome subunits.

| Protein | Gene | Resides with potential $Zn^{2+}$-binding sites | Docking scores | Highest scores and number of iterations |
|---|---|---|---|---|
| α1 | *PSMA6* | 62D, 64S, 65T, 112D, 116K, 137C, 144D, 145E, 146E, 147Q, 154C, 155D, 196E, 234E, 237A, 238H | 2.034-6.007 | 62, 65, 144, 146, 196, 238 |
| α7 | *PSMA3* | 43D, 51K, 60E, 70D, 71R, 72H, 99R, 105N, 144D, 147Q, 188D, 192E, 200V, 201H, 202D, 203E, 206D, 210E, 218E, 233E, 236E, 241E, 245E | 1.958-5.063 | 70-72, 200-206 |
| β1 | *PSMB6* | 38H, 39D, 53Q, 57D, 66H, 69E, 72E, 77H, 78T, 104D, 107E, 145E, 147M, 150E, 151E, 167D, 169S | 2.115-4.728 | 66, 69, 77, 104, 107 |
| β1i | *PSMB9* | 7E, 38H, 39E, 64E, 107E, 122Q, 166D, 168S, 177T, 184D, 185H, 186R | 2.003-4.982 | 7, 38, 39, 107 |
| β5 | *PSMB5* | 115D, 117E, 131G, 150E, 154D, 167D, 169Y, 175N, 190D | 2.065-6.560 | 131, 150, 154, 167, 169 |
| β5i | *PSMB8* | 9Q, 10H, 115D, 116E, 117H, 165H, 191D, 194D, 197H, 201E, | 1.906-4.994 | 115, 117, 197, 201 |

| | | | | |
|---|---|---|---|---|
| RPT4 | *PSMC6* | 101D, 103T, 113R, 114E, 116D, 134E, 140E, 144E, 147E, 173Y, 220N, 224D, 225H, 233D, 234E, 236D, 268D, 271H, 279T, 300H, 316H, 302D, 322K, 323H, 365E, 366D, 369K, 385D, 387K | 1.911-5.272 | 140, 144, 147, 220, 224, 300, 302 |
| RPN3 | *PSMD3* | 36E, 39E, 40E, 50E, 61E, 62H, 186K, 189D, 190D, 194K, 200R, 242H, 265D, 268E, 301E, 304E, 400H, 404K, 418S, 421D, 428L, 429D, 430S, 433D, 440K, 444D | 1.937-8.445 | 50, 61, 62 |
| RPN10 | *PSMD4* | 5S, 11D, 13S, 27Q, 31D, 38H, 41T, 57D, 59E, 94H, 99H, 105H, 119D, 123D, 130R, 133K, 134E, 146E, 147E, 151E | 1.949-3.735 | 11, 13, 38, 41, 57, 59 |
| RPN11 | *PSMD14* | 52E, 83S, 113H, 115H, 120C, 126S, 172H, 173E, 237H, 240H, 241N, 247E, 254N, 279D, 283H, 286E, 287H, 299C | 1.902-4.913 | 52, 113, 115, 126 |

Protein: proteasome subunit symbol; Gene: corresponding gene symbol; Residues with potential $Zn^{2+}$-binding sites: amino acid residues predicted to interact with $Zn^{2+}$, indicated by residue number followed by the one-letter amino acid code (e.g., 62D = aspartic acid at position 62), according to the IUPAC/IUBMB standard nomenclature for amino acids (https://www.qmul.ac.uk/sbcs/iubmb/); Docking scores: range of binding affinity scores obtained from the docking analysis; Highest scores and number of iterations: residues with the strongest docking scores and the number of iterations supporting these predictions.

**Table S2.** Clinical and laboratory characteristics of patients with T1DM stratified by SNPs in *PSMA6*, *PSMB6*, *PSMB9*, *PSMC6*, and *PSMD3* genes.

| **Gene / Polymorphism** | | | |
|---|---|---|---|
| ***PSMA6* / rs1048990 (C/G)** | **C/C** | **C/G + G/G** | **P-value*** |
| HbA1c (%) | 8.7 ± 1.9 | 9.0 ± 2.0 | 0.155 |
| Total cholesterol (mg/dL) | 182.5 ± 44.9 | 185.7 ± 57.0 | 0.550 |
| HDL-C (mg/dL) | 56.5 ± 17.3 | 57.1 ± 16.5 | 0.704 |
| LDL-C (mg/dL) | 105.5 ± 36.8 | 107.7 ± 48.8 | 0.637 |
| Triglycerides (mg/dL) | 79.5 (58.0-123.0) | 83.5 (57.3-119.8) | 0.734 |
| Body Mass Index (kg/m$^2$) | 24.8 ± 3.9 | 24.6 ± 4.0 | 0.603 |
| Systemic arterial hypertension (%) | 38.9 | 35.0 | 0.426 |
| eGFR (ml/min per 1.73 m$^2$) | 91.9 ± 34.1 | 91.7 ± 36.4 | 0.954 |
| Serum Creatinine (µg/dL) | 0.9 (0.8-1.1) | 0.9 (0.7-1.1) | 0.875 |
| Urinary Albumin Excretion (mg/g) | 10.4 (5.0-43-8) | 12.2 (5.0-75.6) | 0.294 |
| Diabetic Kidney Disease (%) | 33.4 | 43.8 | 0.049 |
| Diabetic Retinopathy (%) | 51.3 | 48.9 | 0.660 |
| ***PSMB6* / rs2304975 (C/T)** | **C/C** | **C/T + T/T** | **P-value*** |
| HbA1c (%) | 8.8 ± 2.0 | 9.0 ± 1.8 | 0.636 |
| Total cholesterol (mg/dL) | 183.3 ± 49.0 | 182.1 ± 46.4 | 0.853 |
| HDL-C (mg/dL) | 56.7 ± 17.4 | 57.0 ± 16.5 | 0.888 |
| LDL-C (mg/dL) | 106.1 ± 41.4 | 106.3 ± 39.1 | 0.973 |
| Triglycerides (mg/dL) | 82.0 (57.0-123.0) | 75.0 (60.0-113.0) | 0.623 |
| Body Mass Index (kg/m$^2$) | 24.6 ± 3.9 | 25.0 ± 4.5 | 0.517 |
| Systemic arterial hypertension (%) | 37.5 | 36.3 | 0.926 |
| eGFR (ml/min per 1.73 m$^2$) | 91.4 ± 34.8 | 95.4 ± 34.3 | 0.353 |
| Serum Creatinine (µg/dL) | 0.9 (0.8-1.1) | 0.9 (0.8-1.1) | 0.768 |
| Urinary Albumin Excretion (mg/g) | 11.1 (5.0-48.1) | 9.8 (5.0-111.0) | 0.915 |
| Diabetic Kidney Disease (%) | 37.3 | 36.1 | 0.972 |
| Diabetic Retinopathy (%) | 50.3 | 47.6 | 0.733 |
| ***PSMB9* / rs17587 (G/A)** | **G/G + G/A** | **A/A** | **P-value*** |

| | | | |
|---|---|---|---|
| HbA1c (%) | 8.8 ± 2.0 | 9.5 ± 2.0 | 0.045 |
| Total cholesterol (mg/dL) | 180.9 ± 47.6 | 203.4 ± 62.6 | 0.035 |
| HDL-C (mg/dL) | 56.1 ± 17.0 | 62.5 ±17.6 | 0.030 |
| LDL-C (mg/dL) | 104.9 ± 39.9 | 120.6 ± 56.3 | 0.111 |
| Triglycerides (mg/dL) | 80.0 (57.0-119.8) | 76.0 (57.5-119.0) | 0.908 |
| Body Mass Index (kg/m$^2$) | 24.2 ± 3.6 | 24.0 ± 3.9 | 0.763 |
| Systemic arterial hypertension (%) | 38.2 | 35.0 | 0.816 |
| eGFR (ml/min per 1.73 m$^2$) | 91.4 ± 35.8 | 100.5 ± 26.9 | 0.139 |
| Serum Creatinine (µg/dL) | 0.9 (0.8-1.1) | 0.8 (0.7-1.0) | 0.101 |
| Urinary Albumin Excretion (mg/g) | 10.5 (5.0-61.0) | 12.6 (6.1-22.1) | 0.654 |
| Diabetic Kidney Disease (%) | 39.3 | 27.0 | 0.199 |
| Diabetic Retinopathy (%) | 52.3 | 48.9 | 0.783 |

| ***PSMC6* / rs2295825 (G/C)** | **G/G** | **G/C + C/C** | **P-value*** |
|---|---|---|---|
| HbA1c (%) | 8.9 ± 1.9 | 8.8 ± 2.0 | 0.400 |
| Total cholesterol (mg/dL) | 182.4 ± 46.5 | 184.6 ± 50.8 | 0.629 |
| HDL-C (mg/dL) | 54.4 ± 16.9 | 58.3 ± 16.8 | 0.012 |
| LDL-C (mg/dL) | 105.7 ± 36.3 | 107.1 ± 44.3 | 0.727 |
| Triglycerides (mg/dL) | 89.5 (58.0-136.5) | 75.5 (58.8-110.3) | 0.019 |
| Body Mass Index (kg/m$^2$) | 25.0 ± 4.1 | 24.6 ± 3.8 | 0.324 |
| Systemic arterial hypertension (%) | 42.3 | 34.0 | 0.056 |
| eGFR (ml/min per 1.73 m$^2$) | 92.4 ± 34.9 | 91.5 ± 34.8 | 0.756 |
| Serum Creatinine (µg/dL) | 0.9 (0.8-1.1) | 0.9 (0.8-1.1) | 0.634 |
| Urinary Albumin Excretion (mg/g) | 12.4 (5.0-66.2) | 9.3 (4.9-45.5) | 0.125 |
| Diabetic Kidney Disease (%) | 38.9 | 35.8 | 0.578 |
| Diabetic Retinopathy (%) | 56.5 | 45.3 | 0.009 |

| ***PSMD3* / rs3087852 (G/A)** | **G/G** | **G/A + A/A** | **P-value*** |
|---|---|---|---|
| HbA1c (%) | 8.9 ± 2.0 | 8.8 ± 1.9 | 0.615 |
| Total cholesterol (mg/dL) | 183.4 ± 49.0 | 184.1 ± 48.8 | 0.892 |
| HDL-C (mg/dL) | 54.6 ± 16.4 | 57.3 ± 17.4 | 0.143 |
| LDL-C (mg/dL) | 108.5 ± 40.3 | 105.8 ± 41.1 | 0.527 |
| Triglycerides (mg/dL) | 88.5 (65.0-125.3) | 78.5 (56.0-120.5) | 0.107 |

| | | | |
|---|---|---|---|
| Body Mass Index (kg/m$^2$) | 24.9 ± 4.0 | 24.7 ± 4.0 | 0.751 |
| Systemic arterial hypertension (%) | 40.8 | 36.4 | 0.404 |
| eGFR (ml/min per 1.73 m$^2$) | 94.0 ± 34.7 | 91.3 ± 34.9 | 0.443 |
| Serum Creatinine (µg/dL) | 0.9 (0.8-1.1) | 0.9 (0.8-1.1) | 0.736 |
| Urinary Albumin Excretion (mg/g) | 19.0 (5.0-196.4) | 9.5 (4.8-40.5) | 0.016 |
| Diabetic Kidney Disease (%) | 44.5 | 34.2 | 0.060 |
| Diabetic Retinopathy (%) | 52.3 | 49.3 | 0.590 |

eGFR: estimated glomerular filtration rate; HbA1c: glycated hemoglobin; HDL-C: high-density lipoprotein cholesterol; LDL-C: low-density lipoprotein cholesterol. a Values of variables are presented as percentage, mean ± standard deviation or median (25th-75th percentiles). * P-values were obtained from *t*-test, $\chi^2$ test or Mann-Whitney test according to each variable.
